# The impact of *HLA-DRB1 *genes on extra-articular disease manifestations in rheumatoid arthritis

**DOI:** 10.1186/ar1837

**Published:** 2005-10-11

**Authors:** Carl Turesson, Daniel J Schaid, Cornelia M Weyand, Lennart TH Jacobsson, Jörg J Goronzy, Ingemar F Petersson, Gunnar Sturfelt, Britt-Marie Nyhäll-Wåhlin, Lennart Truedsson, Sonja A Dechant, Eric L Matteson

**Affiliations:** 1 Department of Rheumatology, Malmö University Hospital, Södra Förstadsgatan 101, 205 02 Malmö, Sweden; 2 Division of Rheumatology, Mayo Clinic College of Medicine, 200 First Street SW, Rochester, Minnesota 55905, USA; 3 Department of Health Sciences Research, Mayo Clinic College of Medicine, 200 First Street SW, Rochester, Minnesota 55905, USA; 4 Lowance Center for Human Immunology, Emory University School of Medicine, 101 Woodruff Circle, Atlanta, Georgia 30322, USA; 5 Spenshult Hospital for Rheumatic Diseases, 313 92 Oskarström, Sweden; 6 Department of Rheumatology, Lund University Hospital, Kioskgatan 3, 221 85 Lund, Sweden; 7 Department of Clinical Microbiology and Immunology, Lund University Hospital, Sölvegatan 23, 223 62 Lund, Sweden

## Abstract

The objective of this study was to examine *HLA-DRB1 *and *HLA-DQB1 *genotypes in patients with severe extra-articular rheumatoid arthritis (ExRA) and to compare them with the genotypes of rheumatoid arthritis (RA) patients without extra-articular manifestations. Patients with severe ExRA were recruited from a large research database of patients with RA, from two cohorts of prevalent RA cases, and from a regional multicenter early RA cohort. Cases with ExRA manifestations (*n *= 159) were classified according to predefined criteria. Controls (*n *= 178) with RA but no ExRA were selected from the same sources. Cases and controls were matched for duration of RA and for clinical center. PCR based *HLA-DRB1 *and *HLA-DQB1 *genotyping was performed using the Biotest SSP kit, with additional sequencing in order to distinguish DRB1*04 subtypes. Associations between alleles and disease phenotypes were tested using multiple simulations of random distributions of alleles. There was no difference in global distribution of *HLA-DRB1 *and *HLA-DQB1 *alleles between patients with ExRA and controls. DRB1*0401 (*P *= 0.003) and *0401*/*0401 *homozygosity (*P *= 0.002) were more frequent in Felty's syndrome than in controls. The presence of two HLA-DRB1*04 alleles encoding the shared epitope (SE) was associated with ExRA (overall odds ratio 1.79, 95% confidence interval 1.04–3.08) and with rheumatoid vasculitis (odds ratio 2.44, 95% confidence interval 1.22–4.89). In this large sample of patients with ExRA, Felty's syndrome was the only manifestation that was clearly associated with HLA-DRB1*0401. Other ExRA manifestations were not associated with individual alleles but with DRB1*04 SE double dose genotypes. This confirms that SE genes contribute to RA disease severity and ExRA. Other genetic and environmental factors may have a more specific impact on individual ExRA manifestations.

## Introduction

Rheumatoid arthritis (RA) is a systemic inflammatory disease that, in a substantial proportion of patients, is associated with the development of extra-articular manifestations. These extra-articular RA (ExRA) manifestations can have a defining impact on disease outcome, including increased premature mortality compared with RA in general [1-4]. Severe ExRA occurs both in patients recently diagnosed with RA and in those with long-standing disease [2]. Suggested predictors of ExRA include clinical, serologic, and genetic factors [5].

There is strong evidence of a role for genetic factors in the etiology of RA [6-8], and genetic polymorphisms are probably involved in the wide variation in disease expression. As for most diseases classified according to a list of criteria, rather than specific diagnostic tests, the disease phenotype in RA is heterogeneous. The presence of disease susceptibility alleles may define subsets of patients with different disease courses, including patients with mild, nonerosive disease and those with a true RA phenotype and progressive disease, with extensive joint damage and ExRA manifestations. On the other hand, genetic markers not related to disease susceptibility may influence disease progression and risk for developing ExRA.

*HLA *(human leukocyte antigen) alleles have been implicated in a number of chronic inflammatory diseases. RA has been associated with the 'shared epitope' (SE) of *HLA-DRB1*, which includes DRB1*04 and DRB1*01 alleles [9]. Recent genome-wide scanning studies using microsatellite loci have confirmed that there is strong linkage between this region and RA [10,11]. RA-associated HLA-DRB1*04 alleles have been reported mainly in patients with severe disease [12-16]. A meta-analysis of studies of disease progression in RA [17] revealed an association between HLA-DRB1*04 and erosive disease, and in a recently reported survey of an extensively investigated cohort of patients with early RA [18] homozygosity for HLA-DRB1*04 was a major predictor of development of erosions. DRB1*04 alleles have also been specifically associated with ExRA [19-21], and a specific impact of DRB1*04 homozygosity has been suggested. Some authors have reported an association with the *0401*/*0401 *genotype [21,22] whereas others have found the *0401*/*0404 *genotype to be more frequent among patients with ExRA [23]. These discrepancies may reflect variability in the relative frequencies of HLA-DRB1*0401 in different populations. For example, in East Asian populations, in which DRB1*0401 is rare and DRB1*0405 is the most frequent RA associated *HLA-DRB1 *genotype [24], the latter allele has also been reported to be associated with an increased risk for ExRA manifestations [25].

All previous studies of major histocompatibility class (MHC) class II genes and ExRA have been based on small patient samples, limiting the generalizability of the results. Most studies were not sufficiently powered to examine the effect of linkage disequilibrium within the MHC, including *HLA-DQB1 *alleles. Previous investigations did not use consistent and well characterized definitions of ExRA, which is a matter of vital importance to the study of disease phenotypes in RA [26].

The purpose of this study was to investigate associations between *HLA-DRB1 *and *HLA-DQB1 *alleles and severe ExRA manifestations in a multicenter case-control study of patients with well characterized disease. To our knowledge, this is the largest sample of patients with severe ExRA ever reported. We report that patients with ExRA manifestations are more likely to carry a double dose of DRB1*04 SE alleles, and we demonstrate that the impact of individual *DRB1 *alleles is limited.

## Materials and methods

### Patients

Patients with severe ExRA according to predefined criteria [2,3] were recruited from the rheumatology laboratory database of the Mayo Clinic (Rochester, MN, USA), from two clinic-based cohorts of patients with ExRA from Malmö University Hospital and Lund University Hospital (Sweden), and from a Swedish multicenter early RA cohort (the Better AntiRheumatic PharmacOTherapy [BARFOT] cohort). ExRA manifestations studied included pericarditis, pleuritis, Felty's syndrome, scleritis, episcleritis, glomerulonephritis, vasculitis-related neuropathy, major cutaneous vasculitis, and vasculitis involving other organs. Felty's syndrome was defined as RA-associated neutropenia and splenomegaly, with other potential causes excluded or unlikely. In addition, the criteria for severe ExRA were modified to include RA-associated interstitial lung disease, as previously described [5]. Controls, defined as patients with RA without current or previous signs of extra-articular disease manifestations, in accordance with the same criteria [2,3,5], were selected from the corresponding centers. One patient with RA (control) was matched to each patient with ExRA (case) according to duration of RA and clinical center. All cases and controls fulfilled 1987 American College of Rheumatology criteria for classification of RA [27].

Eighty-eight patients fulfilling the predefined criteria for ExRA (see above) were identified from the Mayo Clinic rheumatology laboratory database, and their medical case records were subjected to a structured review, as previously described [22]. A random sample of 184 patients with RA but without ExRA were identified from this database after careful medical record review. Controls from this sample were matched with cases for duration of RA ± 5 years. DNA samples were available from 86 ExRA cases and 85 controls for *HLA *typing.

Another cohort of patients was recruited from a prospective study of extra-articular disease manifestations and vascular comorbidities in RA from the rheumatology outpatient clinic of Malmö University Hospital. Consecutive patients with recently diagnosed severe extra-articular disease manifestations were invited to participate. Patients with non-extra-articular RA, matched to extra-articular patients for age, sex and disease duration (± 1 year), were selected from a community-based register of RA patients in the city of Malmö [28] or from a community-based early RA inception cohort from the same area. Samples from 28 patients with ExRA (cases) and 28 matched patients with RA but without ExRA (controls) were available for analysis. Thirty-five patients with ExRA (cases) and 42 patients with RA but without extra-articular disease (controls), matched for disease duration, from a case-control study of predictors of ExRA at the University Hospitals in Malmö and Lund [22] were also included in the analysis. Results of *HLA-DR *and *HLA-DQ *genotyping for some of these patients were reported previously [22].

In addition, patients were recruited from the BARFOT registry [29], which includes patients participating in a structured program for follow up of newly diagnosed RA in southern Sweden. This registry includes virtually all adult patients with new onset of inflammatory polyarthritis within the catchment area of the six participating rheumatology centers of the BARFOT program (total population is approximately 1.5 million), including patients fulfilling the 1987 American College of Rheumatology classification criteria for RA [27]. From 1992 to 2001, a total of 1,589 consecutive patients were recruited to the registry. Referring rheumatologists are encouraged to report ExRA manifestations occurring in these patients to the register. All reported ExRA cases (*n *= 35) were reviewed and classified according to the study criteria [2,3]. Of these, 26 patients fulfilled the criteria for ExRA. Controls without ExRA were matched to the cases by sex, age at inclusion, disease duration and, when possible, geographic region. All potential controls were reviewed in order to ensure that they did not have a history of ExRA. Samples for genotyping were available from ten ExRA cases and 24 non-ExRA controls in this subset.

Data on serologic tests for rheumatoid factor (RF) and antinuclear antibodies (ANAs), and information on smoking status are prospectively collected as part of a structured follow up of patients in the BARFOT study. Data on these parameters for patients from the other centers (Malmö, Lund and the Mayo Clinic) were obtained by thorough review of all available clinical records.

All patients gave informed consent to participate in the study. The study was approved by the Research Ethics Committee at Lund University and by the Institutional Review Board at the Mayo Clinic.

### Genotyping

DNA for *HLA-DRB1/DQB1 *typing of patients recruited from the Mayo Clinic was isolated from peripheral blood mononuclear cells using the DNA Isolation Kit for Mammalian Blood (Roche Applied Sciences, Indianapolis, IN, USA). For patients from the Swedish RA cohorts, DNA was extracted from whole blood using the QIAamp minikit (Qiagen, Hilden, Germany) at the DNA/RNA Genotyping Laboratory, SWEGENE Resource Center for Profiling Polygenic Diseases (Lund University and Malmö University Hospital, Sweden). The purified DNA was used for *HLA-DRB1 *and *HLA-DQB1 *determination with the PCR-based Micro-SSP DRB and DQB generic typing trays (Biotest AG, Dreiech, Germany). Because the DRB kit does not detect HLA-DRB1*04 allelic variations, all samples that were positive for HLA-DRB1*04 were re-amplified by PCR using a primer set that amplified all HLA-DRB1*04 alleles: 5'-GTTTCTTGGAGCAGGTTAAACA-3' (HLA-DRB1*04) and 5'-GCCGCTGCACTGTGAAGCTCTC-3' (*HLA-DRB1 *generic). Samples were then purified using the High Pure PCR Product Purification Kit (Roche Applied Sciences) and sequenced in the Mayo Clinic molecular biology core facility on a PRISM 37 DNA Sequencer (Applied Biosystems, Foster City, CA, USA) with the *HLA-DRB1 *primer as the initiating primer. The specific HLA-DRB1*04 allele was then assigned on the basis of the sequencing results. For the statistical analysis, the SE encoding rare DRB1*0401-like alleles *0409, *0413, *0416 and *0421 were classified as *0401; alleles *0408, *0410 and *0419 were classified as *0404. The DRB1*0405 alleles were analyzed as a separate entity. All other DRB1*04 alleles were classified as DRB1*04 non-SE alleles.

### Statistical analysis

The age at RA diagnosis and the duration of RA at inclusion in ExRA cases and non-ExRA controls with RA were compared using the t test. The sex distribution, the number of smokers and the number of patients with a positive RF test or ANA test at any time were compared between the cases and controls using Pearson's χ^2 ^statistic.

To compare the distribution of alleles between cases and controls, we used Armitage's trend in proportions, which does not treat the two alleles within a person as independent (i.e. it does not assume Hardy-Weinberg equilibrium). This approach reduces to the usual Pearson χ^2 ^statistic for comparing allele frequencies when genotype proportions match Hardy-Weinberg proportions [30], and is the preferred way to compare allele frequencies [31]. However, the usual Armitage test for trend is for only two alleles. A multivariate extension for more than two alleles, which compares allele counts between cases and controls, is based on the score statistic for logistic regression. For this score statistic, each subject receives a vector of scores, where each element of the vector counts alleles of each type. From this score statistic, we computed a global test of association between case/control status and all alleles of *HLA-DRB1 *and *HLA-DQB1 *separately. Because the distribution of this statistic is not known, we performed simulations to compute *P *values. The case/control status was randomly permuted, and the simulated statistic computed and compared with the observed statistic. This simulation process was repeated 1,000 times to compute *P *values, both for the maximum statistic and allele-specific Armitage trend tests. The distribution of combinations of *HLA-DRB1 *and *DQ *alleles (i.e. the distribution of *HLA-DRB1-DQ *haplotypes) was similarly compared in cases and controls.

To evaluate the association of single or double dose of HLA-DRB1*04 SE subtypes with case/control status, or with a particular manifestation of ExRA, we used logistic regression to calculate odds ratios (ORs) and 95% confidence interval (CI).

## Results

A total of 159 patients with severe ExRA according to predefined criteria [5,23] were identified. Forty-three patients had vasculitis, defined as biopsy proven vasculitis or major cutaneous vasculitis diagnosed by a dermatologist. Additional subgroups analyzed were neuropathy (mononeuropathy or polyneuropathy; *n *= 40), interstitial lung disease (*n *= 25), Felty's syndrome (*n *= 21) and pericarditis (*n *= 27). These were compared with 178 controls with non-extra-articular RA. Disease duration and age at RA onset was similar in cases and controls (mean 11.3 years versus 12.5 years for duration, and mean 50.1 years versus 50.4 years for age at RA onset; Table 1). There was a trend toward a relative predominance of male patients in the ExRA group (*P *= 0.06). However, this comparison is skewed because of the matching of cases and controls for sex in two of the subsamples. A positive test for RF (*P *< 0.0001) or ANAs (*P *< 0.0001) at any time were both significantly associated with ExRA.

Some of the individual severe ExRA manifestations occurred together more frequently than expected. Among the 21 patients with Felty's syndrome, three (14%) had evidence of vasculitis. In the subset with vasculitis, 15 out of 43 (35%) had neuropathy and seven (16%) had interstitial lung disease.

### Overall effects of *HLA*-*DRB1 *alleles

**Figure 1:**
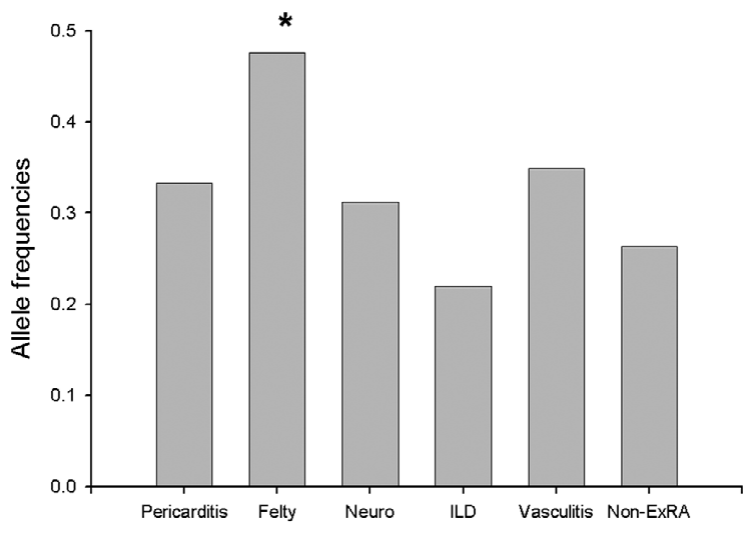
Variation in frequency of HLA-DRB1*0401 by disease phenotype in RA. HLA-DRB1*0401 was significantly more frequent in patients with Felty's syndrome (*P *= 0.003) than in non-extra-articular rheumatoid arthritis (RA) controls, but patients with other manifestations did not differ significantly from controls. ExRA, extra-articular RA; ILD, interstitial lung disease; Neuro, vasculitis related neuropathy.

**Figure 2:**
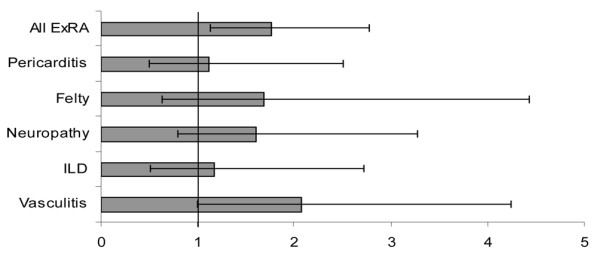
ExRA manifestations among those carrying carrying HLA-DRB1*04 shared epitope alleles. Shown are odds ratios (ORs) with 95% confidence intervals (CIs) for different extra-articular rheumatoid arthritis (ExRA) manifestations for patients carrying HLA-DRB1*04 shared epitope alleles. ILD, interstitial lung disease.

The distribution of *HLA-DRB1 *was not significantly different between ExRA cases and non-ExRA controls (global *P *= 0.19; Table 2). The most frequent *HLA-DRB1 *allele in both groups was HLA-DRB1*0401, and this allele tended to be more common among patients with ExRA (allele frequency 0.326 versus 0.263; *P *= 0.09). HLA-DRB1*0401 was significantly associated with Felty's syndrome (allele frequency 0.475; *P *= 0.003) but not with other individual manifestations when compared with non-extra-articular RA (Fig. 1). The rare allele HLA-DRB1*12 was more common in the ExRA subgroup (allele frequency 0.023 versus 0.003; *P *= 0.02). The DRB1*0405 (allele frequency 0.019 versus 0.003; *P *= 0.01) and DRB1*0404 (allele frequency 0.119 versus 0.085; *P *= 0.14) alleles were also more frequent in patients with ExRA than in non-ExRA controls. One of the HLA-DRB1*04 alleles encoding the SE (DRB1*0401, *0404, or *0405) was present in 105 out of 151 ExRA patients as compared with 96 out of 178 non-ExRA patients (OR 1.77, 95% CI 1.13–2.77). The impact of the presence of DRB1*04 SE alleles on risk for ExRA was variable for the different manifestations (Fig. 2). Patients with RA and vasculitis were more likely to carry DRB1*04 SE alleles than patients with RA and no vasculitis (OR 2.07, 95% CI 1.00–4.25). Similar trends were found for Felty's syndrome and neuropathy, but the associations were not significant (Fig. 2).

### Effects of homozygosity for the shared epitope

The homozygous genotype HLA-DRB1*0401/0401 was significantly more frequent in patients with Felty's syndrome (genotype frequency 0.286; *P *= 0.002) and patients with pericarditis (genotype frequency 0.185; *P *= 0.043) than in non-ExRA controls (frequency 0.068). Other ExRA manifestations were not associated with any specific homozygous genotype. The presence of two HLA-DRB1*04 SE alleles was significantly associated with ExRA overall (OR 1.79, 95% CI 1.04–3.08), Felty's syndrome (OR 2.63, 95% CI 1.04–6.63), and vasculitis (OR 2.44, 95% CI 1.22–4.89) compared with patients with RA who lacked these manifestations. By contrast, pericarditis, neuropathy, and interstitial lung disease were not associated with double dose of HLA-DRB1*04 SE alleles (Table 3).

### Effects of *HLA-DQB *alleles

The distribution of *HLA-DQB *alleles was not significantly different between ExRA cases and non-ExRA controls (*P *= 0.11; Table 4). The relatively rare allele *HLA-DQ4 *tended to occur more frequently in ExRA cases (allele frequency 0.046 versus 0.014; *P *= 0.037). Other than that, there was no significant difference in the occurrence of *DQB *alleles between patients with ExRA overall or individual ExRA manifestations and non-ExRA controls. There was no significant global difference in the frequency of homozygous *HLA-DQB *genotypes between cases and controls except for patients with ExRA and pericarditis (*P *= 0.04). *HLA-DQ8/DQ8 *homozygosity was more common in patients with pericarditis than in non-ExRA patients with RA (genotype frequency 0.120 versus 0.029; *P *= 0.021).

### Analyses of linkage disequilibrium

Haplotype analysis indicated that the association between ExRA and HLA-DRB1*04 SE homozygosity was due to the importance of the DRB1*04 genotype, rather than being secondary to associations with *HLA-DRB1-DQB *haplotypes (data not shown). Similarly, the associations between Felty's syndrome and DRB1*0401, and between pericarditis and *DQ8/DQ8 *were not explained by *DRB1-DQB *haplotype associations.

## Discussion

In this large sample of patients with severe ExRA, we found Felty's syndrome to be associated with HLA-DRB1*0401. There was no significant difference in the global distribution of *HLA-DRB1 *or *HLA-DQB *alleles compared with non-extra-articular RA controls for any other manifestation or for ExRA overall. Patients with severe ExRA were more likely to carry HLA-DRB1*04 SE alleles, and genotypes featuring a double dose of DRB1*04 SE alleles were associated with rheumatoid vasculitis, Felty's syndrome, and all ExRA combined.

A number of studies have indicated a role for *HLA-DR4 *related genes in ExRA [26]. In Caucasians of Northern European origin, severe ExRA has been associated with DRB1*0401/0401 homozygosity in particular [21,22]. In a recent meta-analysis of *HLA-DRB1 *genotyping studies of patients with RA-associated vasculitis conducted by Gorman and coworkers [32], vasculitis was found to be associated with the genotypes *0401/0401*, *0401/0404*, and *0401/0101*. In other meta-analyses by the same group, double dose of DRB1*04 SE alleles was associated with radiographic signs of progressive joint damage in northern European Caucasians [17], but there was no significant association between SE and the presence of rheumatoid nodules [33]. Taken together, these findings indicate that DRB1*04 SE double gene dose is associated with disease severity in RA, and that such genotypes may contribute specifically to risk for severe ExRA manifestations.

On the other hand, there was considerable heterogeneity across individual ExRA manifestations. The association between Felty's syndrome and DRB1*0401 is well established [34,35]. In contrast, we did not observe any significant association with single or double DRB1*04 gene dose for patients with pericarditis, neuropathy, or interstitial lung disease. This indicates that the importance of *HLA-DRB1 *alleles may be variable for different manifestations, although our failure to detect an effect could be due to sample size or selection.

Severe ExRA manifestations tend to cluster in individual patients with RA [36]. The high prevalence of vasculitis in patients with Felty's syndrome observed in the present study is consistent with the literature [37], and may in part be due to shared genetic factors such as HLA-DRB1*04 alleles. In a survey of the community-based Olmsted county RA cohort [36] we found clustering of a number of different ExRA features, including a frequent co-occurrence of vasculitis with neuropathy and rheumatoid lung disease. We made similar observations in the present study. Such clustering may be explained by both genetic and environmental factors.

The association between *HLA-DRB1 *genotypes and RA disease severity, including ExRA, has been interpreted as reflecting the importance of T cells in the pathogenesis of RA [26]. HLA-DR and other MHC molecules are involved in presentation of antigens to T cells, and in positive and negative selection of T cells in the thymus. Because there appears to be a stoichiometric relationship between MHC molecules on the cell surface and positive selection mechanisms in thymic maturation of T cells, it has been suggested that the explanation for the gene dose effect seen in ExRA is its effect on T-cell diversity [21,38]. The T-cell repertoire in patients with RA is markedly contracted, with less diversity and emergence of dominant T-cell clonotypes [39]. T-cell abnormalities in patients with ExRA include expansion of CD8^+ ^large granular lymphocytes [40] and of immunosenescent CD4^+^CD28^- ^cells [41,42], and extensive CD4^+ ^infiltrates in RA-associated interstitial pneumonitis [43]. The importance of *HLA-DRB1 *genes and other genes with a role in T-cell selection and T-cell function for these phenomena require further study.

In accordance with previous studies, we found patients with ExRA to be more likely to be RF positive and ANA positive [22,44]. This suggests a role for both B cells and T cells, possibly including dysregulated B cell-T cell interaction, in ExRA.

New genetic associations that were not postulated and have not been reproduced should be interpreted with caution. Given the nonsignificant results of the global distribution tests, the associations between ExRA and some rare *DRB1 *and *DQB1 *alleles (i.e. DRB1*12 and *DQ4*) are probably due to chance. The negative global test for *HLA-DRB1 *alleles in ExRA overall also suggests that the impact of DRB1*04 SE on the risk for severe ExRA manifestations is not strong, although it is reproducible in separate patient samples.

The lack of association between ExRA and *HLA-DQB1 *alleles, and the lack of association with *HLA-DRB1-DQB1 *haplotypes favors a specific role for *HLA-DRB1 *genes in ExRA, rather than secondary associations due to linked genes. Nevertheless, we cannot exclude the possibility that linkage disequilibrium with other genes in MHC explain our results.

The patients included in this study were recruited from four different centers, and the background RA population from which they were sampled is not fully characterized, at least not for the patients seen at Lund University Hospital and at the Mayo Clinic. On the other hand, these patients were recruited during a period when there was particular interest in patients with severe ExRA at each of the centers, suggesting that they should reflect the majority of patients with ExRA seen and be representative of the ExRA population as a whole.

In multicenter studies of genetic markers, ethnic heterogeneity of the studied patient samples must be considered. However, the majority of the patients included at the Mayo Clinic were Caucasians of northern European origin, similar to the patients from southern Sweden. Thus, our result could be generalized to RA patients with this ethnic background but not to other populations.

## Conclusion

In a study of a large sample of patients with ExRA, we have confirmed an association between HLA-DRB1*0401 and Felty's syndrome, but we found no association between ExRA overall or other individual manifestations and specific *HLA-DRB1 *alleles. Double dose HLA-DRB1*04 SE genotypes are associated with a modestly increased risk for vasculitis and other ExRA manifestations. Other genetic and environmental factors are likely to be more important for the systemic features of RA.

## Abbreviations

ANA = antinuclear antibody; CI = confidence interval; ExRA = extra-articular rheumatoid arthritis; HLA = human leukocyte antigen; MHC = major histocompatibility complex; OR = odds ratio; PCR = polymerase chain reaction; RF = rheumatoid factor; SE = shared epitope.

## Competing interests

The authors declare that they have no competing interests.

## Authors' contributions

CT conceived the study, was responsible for the recruitment and classification of patients, and drafted the manuscript. DS performed the statistical analysis and helped to draft the manuscript. CW participated in the design and coordination of the study, and recruited a subset of the patients. LJ participated in the recruitment of a subset of the patients and the interpretation of the statistical data, and helped to draft the manuscript. JG recruited a subset of the patients and participated in the design and coordination of the study. GS participated in the recruitment of patients and the molecular genetics analysis. IP participated in the recruitment of patients, the extraction of clinical data, and the interpretation of the statistical analysis. BMNW participated in the recruitment and classification of patients and the extraction of clinical data. LT performed part of the molecular genetics analysis and helped to draft the manuscript. SD performed part of the molecular genetics analysis and participated in the coordination of the study. EM conceived the study together with CT, performed part of the molecular genetics analysis, participated in the design and coordination of the study, and in the interpretation of the statistical data, and helped to draft the manuscript. All authors read and approved the final manuscript.

## Figures and Tables

**Table 1:** Demographic data and clinical predictors of ExRA

	ExRA	Non-ExRA	*P*
Number	159	178	
Age at RA diagnosis (years; mean ± SD)	50.1 ± 14.4	50.4 ± 14.8	0.87
Disease duration (years; mean ± SD)	11.3 ± 11.2	12.5 ± 11.3	0.34
Male/female (*n*)	75/84	66/112	0.06
RF positive^a ^(%)	87.2	58.3	<0.0001
ANA positive^b ^(%)	60.8	33.8	<0.0001

^a^Information available for 149 extra-articular rheumatoid arthritis (ExRA) and 163 non-ExRA patients.

^b^Information available from 120 ExRA and 151 non-ExRA patients. ANA, antinuclear antibody; RA, rheumatoid arthritis; RF, rheumatoid factor; SD, standard deviation.

**Table 2:** Frequencies of *HLA-DRB1 *alleles in patients with ExRA compared with patients with non-extra-articular RA

*HLA-DRB1 *allele	Allele frequency		*P*
		
	ExRA	Non-ExRA	
DRB1*01	0.119	0.130	0.74
DRB1*03	0.071	0.113	0.12
DRB1*0401	0.326	0.263	0.09
DRB1*0404	0.119	0.085	0.14
DRB1*0405	0.019	0.003	0.01
Non-SE DRB1*04	0.019	0.045	0.09
DRB1*07	0.052	0.065	0.54
DRB1*08	0.013	0.014	1.00
DRB1*09	0.023	0.011	0.23
DRB1*10	0.016	0.020	0.73
DRB1*11	0.036	0.054	0.28
DRB1*12	0.023	0.003	0.02
DRB1*13	0.042	0.045	0.83
DRB1*15	0.097	0.110	0.65

Global: *P *= 0.19. ExRA, extra-articular rheumatoid arthritis; SE, shared epitope.

**Table 3:** The effect of homozygosity for HLA-DRB1*04 shared epitope alleles on risk for severe ExRA manifestations

Manifestations	Homozygotes (yes/no; %)		OR (95% CI)	*P*
			
	Cases with manifestation	Controls without manifestation		
All ExRA	39/116 (25)	28/149 (16)	1.79 (1.04–3.08)	0.04
Pericarditis	8/19 (29)	59/246 (19)	1.76 (0.73–4.19)	0.21
Felty's syndrome	8/13 (38)	59/252 (19)	2.63 (1.04–6.63)	0.04
Neuropathy	8/32 (20)	59/233 (20)	0.99 (0.43–2.25)	0.98
Interstitial lung disease	3/22 (12)	64/243 (21)	0.52 (0.15–1.78)	0.30
Vasculitis	15/28 (35)	52/237 (18)	2.44 (1.22–4.89)	0.01

CI, confidence interval; ExRA, extra-articular rheumatoid arthritis; OR, odds ratio.

**Table 4:** Frequencies of *HLA-DQ *alleles in patients with ExRA compared with patients with non-extra-articular RA

*HLA-DQ *allele	Allele frequency		*P*
		
	ExRA	Non-ExRA	
*DQ2*	0.122	0.121	0.91
*DQ3*	0.017	0.029	0.50
*DQ4*	0.046	0.014	0.04
*DQ5*	0.129	0.188	0.06
*DQ6*	0.139	0.165	0.37
*DQ7*	0.252	0.249	1.00
*DQ8*	0.222	0.170	0.13
*DQ9*	0.066	0.043	0.22

Global: *P *= 0.11. ExRA, extra-articular RA.
